# Determination of polycyclic aromatic hydrocarbons in bud-derived supplements by magnetic molecular imprinted microparticles and GC-MS: D-optimal design for a fast method optimization

**DOI:** 10.1038/s41598-023-44398-8

**Published:** 2023-10-16

**Authors:** Barbara Benedetti, Arianna Tronconi, Federica Turrini, Marina Di Carro, Dario Donno, Gabriele Loris Beccaro, Raffaella Boggia, Emanuele Magi

**Affiliations:** 1 Department of Chemistry and Industrial Chemistry, University of Genoa, Via Dodecaneso 31, 16146 Genoa, Italy; 2 Present Address: Paul Scherrer Institut, Forschungsstrasse 111, Villigen, 5303 Switzerland; 3 Department of Pharmacy, University of Genoa, Viale Cembrano 4, 16148 Genoa, Italy; 4 Department of Agriculture, Forestry and Food Science, University of Turin, Largo Braccini 2, 10095 Grugliasco (TO), Italy

**Keywords:** Analytical chemistry, Mass spectrometry

## Abstract

Within the world of natural food supplements, organic extracts deriving from young plant meristematic tissue (bud-derivatives) are becoming attractive, thanks to their richness in bioactive molecules. This natural source is scarce, but every year, tons of plant material, including buds, come from city pruning. If this sustainable source is rather promising from a circular economy point of view, the safety of the obtained supplements must be assessed. In fact, anthropic microcontaminants, such as polycyclic aromatic hydrocarbons (PAHs), could adsorb onto the urban buds, leading to a possible contamination of the bud-derivatives. In this study, we developed a magnetic dispersive solid phase extraction (m-dSPE) based on molecularly imprinted microparticles, combined with GC-MS, to quantify the 16 priority PAHs in such extracts. The D-optimal experimental design was implemented to maximize analytes’ recovery with the smallest set of experiments. The optimized method was characterized by great selectivity thanks to the molecular imprinted polymer and ease of use provided by m-dSPE. Moreover, it complies with green principles, thanks to the minimum consumption of organic solvent (1.5 mL of acetone per sample). The recoveries ranged from 76 to 100% and procedural precision was below 10% for most PAHs. Despite the matrix complexity, low quantification limits (0.7–12.6 μg kg^−1^) were reached. This guaranteed the PAHs’ quantitation at levels below those indicated as safe by a European Community regulation on food supplements. None of the analyzed samples, coming from different anthropically impacted areas, showed concerning PAHs levels.

## Introduction

The use of plant-derived food supplements has greatly increased in the latest years, also due to the growing belief that “natural is better, healthier and safer” than synthetic drugs^1^. Among the several natural sources employed for the production of plant food supplements, an option is represented by buds or young sprouts. These represent the young meristematic fresh tissues of tree species which turn into leaves, flowers or fruits, namely the corresponding plant adult parts^2,3^. Although health benefits have not yet been officially approved by the European Food Safety Authority (EFSA), the use of this plant material is well known in traditional medicine and called “gemmotherapy”^4^. Indeed, bud meristematic tissues are very rich in bioactive molecules, such as plant hormones, terpenes, organic acids, vitamins, and in particular polyphenols^5,6^. Several studies focused on different factors that may influence the contents of these health-promoting compounds in plant materials and bud-derived preparations, such as genotype, phenological stages, pedoclimatic conditions, used agrotechniques as well as extraction methods and processing procedures^7,8^.

To provide the optimal content of such molecules, buds must be collected in a rather small period during the year, corresponding to the annual germination of the plant (late winter/early spring)^6^. Therefore, limited amounts of this raw material are available throughout the year in spontaneous trees; moreover, a complete depletion of the trees from buds should be avoided, thus further reducing the obtainable material.

A potential alternative source could be represented by city pruning. Every year, tons of plant material is cut from urban trees and considered as waste, ignoring its potential value^9,10^. Instead, the 3-R principle of circular economy (reduce, reuse and recycle) conveys a much more rational exploitation of environmental resources^11^. This objective involves the recovery of molecules of interest from by-products or waste, to generate benefits for several productive sectors^9,12^.

To obtain nutraceutical products from buds (bud derivatives, BDs), the conventional cold maceration and the innovative Ultrasound-Assisted Extraction (UAE) can be employed, by using a mixture of water, glycerine and ethanol as extraction solvents, according to the European Pharmacopeia 8th edition (2014), following the procedure deriving from the French Pharmacopeia^13,14^.

If it is true that plant-derived extracts can positively affect human health, thanks to the high concentration of bioactive compounds, their natural feature does not intrinsically guarantee safety. In the case of “urban” buds, contamination by anthropic pollutants can occur, due to particulate matter deposition on them. Among potential contaminants, the presence of polycyclic aromatic hydrocarbons (PAHs) in food supplements is regulated by the Regulation 915/2023^15^. PAHs are a class of well-known persistent organic pollutants, which generate concern due to their toxicity and ubiquitous presence in the environment^16,17^. Sixteen of them are considered priority pollutants according to the United States Environmental Protection Agency (US EPA) and 7 are classified as probable carcinogenic (benzo[a]anthracene, chrysene, benzo[b]fluoranthene, benzo[k]fluoranthene, benzo[a]pyrene, indeno[1,2,3-c,d]pyrene and dibenzo[a,h]anthracene). Consequently, verifying the compliance with the EC regulation becomes essential to guarantee the safety of BDs for human consumption.

Due to the complexity of the supplement composition, a specific analytical method is required to detect and accurately quantify PAHs. In this context, the exploitation of molecular imprinted polymers (MIPs) represents a valuable option. In fact, MIPs are considered as “synthetic receptors” for the high selectivity brought by the cavities present in the bulk polymer^18,19^. A powerful combination in terms of innovative analytical pre-treatment is constituted by magnetic microparticles covered with MIPs (MagMIPs), which brings significant advantages compared to the conventional solid phase extraction, such as low solvent consumption, reusability and ease of use^20,21^. MagMIPs have been successfully employed to extract PAHs from environmental matrices, such as freshwater and seawater^22–26^, nevertheless, their application to food supplements has never been reported.

In this context, an accurate analytical procedure was developed to investigate the presence of the 16 priority PAHs in *Tilia tomentosa* BDs, obtained by maceration or UAE of the buds in a mix of glycerin:ethanol. The PAHs’ extraction by MagMIP methodology was optimized through the application of D-optimal design and gas chromatography-mass spectrometry (GC-MS) was exploited for the analysis. The final method was successfully applied to quantify the 16 pollutants in derivatives obtained from buds coming from regions with different anthropic impacts.

## Materials and methods

### Chemicals and reagents

A standard solution containing the 16 priority PAHs dissolved in acetone/benzene at a concentration of 2000 µg mL^−1^ each, was purchased from Dr. Ehrenstorfer GmbH (Augsburg, Germany). The considered analytes were the following: naphthalene (NAP), acenaphthylene (ACL), acenaphthene (AC), fluorene (FL), phenanthrene (PH), anthracene (ANT), fluoranthene (FLT), pyrene (PY), benzo[a]anthracene (BaA), chrysene (CHR), benzo[b]fluoranthene (BbF), benzo[k]fluoranthene (BkF), benzo[a]pyrene (BaPY), indeno[1,2,3-c,d]pyrene (IcdPY), dibenzo[a,h]anthracene (DahA) and benzo[g,h,i]perylene (BghiPE). A standard solution containing 5 completely deuterated PAHs in toluene at a concentration of 1000 µg mL^−1^ each, was purchased from Dr. Ehrenstorfer GmbH (Augsburg, Germany). The deuterated compounds were d- naphthalene (d-NAP), d-acenaphthene (d-AC), d-phenanthrene (d-PH), d-chrysene (d-CHR) and d-perylene (d-PE). Pharmaceutical grade ethanol 96% and glycerin were purchased from Sigma-Aldrich (St. Louis, MO, USA). Chromatographic grade acetone and ethanol were from VWR Chemicals (Fontenay-sous-Bois, France). Ultra-pure water was obtained by a Millipore Q-Gard system equipped with a Millipak 0.22 µm filter (Millipore, Watford, Hertfordshire, UK).

Magnetic microparticles covered with a molecular imprinted polymer (MagMIP) were provided by NanoMyp (Granada, Spain). The particles (average diameter of 3 µm) were constituted of a magnetite core (γ-Fe_3_O_4_) covered by a cross-linked vinylic polymer. The template molecule used for the imprinting of the MagMIP was pyrene.

### Instrumentation and GC-MS analysis

The analyses were performed by a 7890A gas chromatograph coupled to a 5975C MSD mass spectrometer, both from Agilent (Agilent Technologies, Santa Clara, USA), and equipped with a Gerstel MPS MultiPurpose Sampler (Gerstel GmbH & Co.KG, Mülheim an der Ruhr, Germany). Chromatographic separation was achieved in a 36 min run by employing a Rxi-5Sil MS capillary column (30 m × 0.25 mm ID, 0.25 µm film thickness) from Restek Italy (Cernusco sul Naviglio, Italy). The injector was set at 250 °C and the injection volume was 1 µL (splitless mode). The carrier gas was helium at a flow rate of 1.2 mL min^−1^ and the following temperature program was used: initial temperature of 60 °C, (held constant for 2 min); first ramp to 280 °C at a rate of 10 °C min^−1^ (held for 5 min); second ramp to 300 °C at 10 °C min^−1^, kept constant for 10 min. An electron impact source in positive ionization mode (EI +) was employed with an electron energy of 70 eV. The transfer line and ion source temperatures were set at 280 °C and 230 °C, respectively. The mass analyzer was a single quadrupole operating in single ion monitoring (SIM) mode: the m/z values corresponding to the molecular ion (M^+.^) of each PAH (analytes and internal standards) are shown in Table S1 (Supplementary Information). BbF and BkF gave partially overlapped chromatographic peaks; thus, they were quantified as the sum. The Agilent GC/MSD MassHunter Acquisition Software and the MassHunter MSD Data Analysis software were used for data acquisition and processing, respectively.

### D-optimal experimental design

A D-optimal design was performed for the procedure optimization^27^. The response to be maximized was the PAH recovery. The following variables were taken into account:

- sample volume/microparticles amount ratio (V_matrix_/m_µp_),
- number of extractions (on the same volume of sample with the same weighted m_µp_)
- extraction time
- extraction mode (type of agitation).

A different number of levels was selected for each variable, also considering their nature (qualitative, semi-quantitative or quantitative), and are presented in Table 1. All possible combinations of the variables’ levels were considered to create the matrix of candidate experiments (n = 36), to be used for the D-optimal implementation.

**Table 1:** Factors investigated by the D-optimal design: type, levels and real values.

	Factor	Variable type	Coded levels	Real values
X_1_	V_matrix_/m_µp_	quantitative	− 1	0.4 mL/mg
			0	1.2 mL/mg
			1	2 mL/mg
X_2_	Extraction time	quantitative	− 1	15 min
			0	30 min
			1	45 min
X_3_	Extraction mode	qualitative	− 1	Soft^a^
			1	Hard^b^
X_4_	Number of extractions	semi-quantitative	− 1	1
			1	2

^a^Soft = gentle rotary agitation.

^b^Hard = ultrasonic agitation.

The Federov exchange algorithm was used to select the best subset among all the possible experiments. The choice of how many and which of the candidate experiments to perform was based on a compromise between the highest normalized determinant of the information matrix and the quality of the design (evaluation of the maximum Variance Inflation Factors). The 18 experiments reported in Table 2 were those selected, plus 3 replicated points, chosen by applying the “D-optimal by addition” method. The latter algorithm allows to select the most informative experiments among the selected ones, thus suggesting which are the best to be carried out in replicate. Performing replicated experiments is of fundamental importance to estimate the variance of the procedure and make the interpretation of results more reliable. To summarize, a total of 21 experiments were performed to allow the computation of the following mathematical model (13 coefficients):

1 $$\begin{array}{c}Y={b}_{0}+{b}_{1}{x}_{1}+{b}_{2}{x}_{2}+{b}_{3}{x}_{3}+{b}_{4}{x}_{4}+{b}_{11}{x}_{1}^{2}+{b}_{22}{x}_{2}^{2}+\\ {+ b}_{12}{x}_{1}{x}_{2}+{b}_{13}{x}_{1}{x}_{3}+{b}_{14}{x}_{1}{x}_{4}+{b}_{23}{x}_{2}{x}_{3}+{b}_{24}{x}_{2}{x}_{4}+{b}_{34}{x}_{3}{x}_{4}\end{array}$$

where *Y* is the response (PAH recovery), *b*_*i*_ are the coefficients of the linear terms, *bii* are the coefficients of the quadratic terms and *bij* are the coefficients of the interactions.

**Table 2:** Selected experiments for the D-optimal design.

Experiment	X_1_	X_2_	X_3_	X_4_
1	0	1	− 1	− 1
2	− 1	− 1	1	− 1
3	1	− 1	1	− 1
4	0	0	1	− 1
5	− 1	1	1	− 1
6	1	1	1	− 1
7	− 1	− 1	− 1	1
8	1	− 1	− 1	1
9	0	0	− 1	1
10	− 1	1	− 1	1
11	1	1	− 1	1
12	− 1	− 1	1	1
13	1	− 1	1	1
14	− 1	1	1	1
15	1	1	1	1
16	0	− 1	− 1	− 1
16 replicate 2	0	− 1	− 1	− 1
17	− 1	0	− 1	− 1
17 replicate 2	− 1	0	− 1	− 1
18	1	0	− 1	− 1
18 replicate 2	1	0	− 1	− 1

The open-source software CAT^28^ was employed to implement the D-optimal design and for computation of the response surface models.

### Bud-derivatives

*Tilia tomentosa* Moench fresh buds were collected during the spring of 2019 and 2021, in 3 urban and sub-urban areas of the city of Turin (Italy), with different degrees of environmental pollution. Table S2 (Supplementary Information) provides the main information on the 9 considered samples (S1–S9). Buds were immediately processed to prepare the corresponding BDs to minimize any degradation preserving the specific composition in bioactive compounds (phytocomplex) as much undamaged as possible. The manufacturing step was performed following two different preparation methods: the conventional cold maceration described for glyceric macerates^14^ and the innovative, faster, UAE recently described by the Authors^5^. Moreover, for both preparation methods the same extraction solvent (a mixture of ethanol and glycerin) and the same bud/solvent ratio (1/20 considering the dry weight) were used, according to the European Pharmacopeia. Each extraction process was performed in duplicate.

### Final extraction procedure

The BDs (extracts or macerates) were centrifuged to remove any solid residue. The supernatant was diluted with ultrapure water in a proportion of 3:1, (v/v) and 15 µL of an internal standard mix at a concentration of 50 mg L^-1^ were added to 1 mL of the diluted solution. This sample was put into a vial containing 10 mg of MagMIP, previously dispersed in 50 µL of acetone, to avoid aggregation. The samples were placed in an ultrasonic bath for 30 min and then a magnet was used to separate the solution from the microparticles. The solution was recovered and kept for the second extraction, while the microparticles were dispersed in 50 μL of EtOH, washed three times with 1 mL of water and let dry. Then, they were subjected to a double “back extraction” with 375 µL of acetone (each). The procedure was repeated on the recovered solution, leading to a total final volume of 1.5 mL. The samples were filtered by a 0.22 µm polytetrafluoroethylene filters and 1 µL was injected for the GC-MS analysis.

### Method performances

The overall method involving the optimal procedure combined with the instrumental analysis was evaluated in terms of linearity range, limits of detection (LODs) and quantitation (LOQs), procedural precision, specificity and accuracy. Calibration curves were built by analyzing standard solutions in acetone at different concentrations, ranging from 1 to 50 μg L^−1^, to investigate the linearity range. The internal standard method was applied for quantitation, thus area ratios among each PAH and the assigned deuterated internal standard were used to build calibration curves. LODs and LOQs in matrix were calculated as the concentrations providing signal-to-noise (S/N) ratio values of 3 and 10, respectively, considering the noise signal in real extracts. These values were expressed as μg kg^−1^, by taking into account all dilution factors and BDs density. Intra-day and inter-day instrumental precision were assessed at the concentration levels of 4 and 50 μg L^−1^, with six replicates per level (intra-day) and during three non-consecutive days (inter-day). The precision of the whole analytical procedure, including sample preparation and GC-MS analysis was evaluated by performing triplicate recovery tests over two non-consecutive days. Accuracy is related to both matrix effects (ME) and recovery of the extraction procedure. As far as ME is concerned, the use of proper internal standards (five in total) covering the whole mass range of the 16 PAHs, allowed to take into account possible variations of the signals due to interferences in the real samples (possibly causing signal suppression or enhancement). Indeed, the ionization efficiency of the non-deuterated and deuterated standards are supposed to be influenced in the same way. Regarding recovery of the final procedure^29^ it was calculated by the following expression:

$$R (\%)= 100* \frac{{A}_{SB}}{{A}_{SA}}$$

where *A*_*SB*_ represents the peak area of the analytes in the sample spiked before processing (SB sample) and *A*_*SA*_ represents the peak area of the analytes in the sample spiked after processing (SA sample), the latter corresponding to a theoretical 100% recovery sample.

Finally, the developed method was evaluated in terms of greenness, by using the recent metrics tools “AGREE”^30^ and “AGREEprep”^31^.

## Results and discussion

### Preliminary tests

In all tests performed for method development a blank matrix constituted by EtOH:Glycerin (1:1, w/w) was used, spiked with a known amount of PAHs. The fortification corresponded to a theoretical PAH 100%-recovery concentration of 100 μg L^−1^ in the final extract. This simplified sample resembled the viscosity and main composition of the BDs, making it suitable for the optimization of the sample-preparation.

Compared to the real samples, the blank matrix was free from any interferent compound, thus no matrix effect was expected. Therefore, during these preliminary tests, internal standards were not employed, and recovery was considered equal to process efficiency^32^:

$$R (\%)=PE\left(\%\right)= 100* \frac{{A}_{SB}}{{A}_{STD}}$$

where A_SB_ and A_STD_ represent the chromatographic peak area of each PAH in the spiked blank and in the 100 μg L^−1^ neat standard, respectively.

The first tests involved the use of a previous protocol based on MagMIP, developed for the quantitation of PAHs in seawater^22^. Briefly, this procedure involved the use of a V_matrix_/m_µp_ ratio of 1 (1 mg of MagMIP for 1 mL of BD), agitation of the sample for 15 min, recovery of the microparticles by using a magnet and PAHs’ back extraction with 150 μL of acetone. The results obtained by the direct application of the method were not satisfactory, with rather low recoveries for “light” PAHs. In fact, R% in the range 3–27% were obtained for PAHs with molecular mass up to 202 Da and 23–61% for PAHs with molecular mass from 228 to 278 Da. Moreover, an important issue arose, related to the glycerin presence in the matrix. Due to its viscosity, traces of glycerin tended to stick to the microparticles, and a rather intense peak was detected by the mass spectrometer and attributed to a glycerin-related product (mass spectrum and NIST library identification are reported as Fig. S1 in supplementary information). Both these results highlighted the substantial different behavior of the MIP in the BD matrix, which made necessary a careful optimization.

First, a washing step was added to completely eliminate glycerin, which would be useful also to eliminate other interferents from the real samples, adsorbed onto the MagMIP particles through non-specific interactions. Three protocols with different washing solvents were tested, the first involving H_2_O:EtOH (1:1, v/v), the second pure H_2_O and the third H_2_O with minimal % of EtOH. In each protocol all the other steps were kept constant and three washings, each with 1 mL of solvent, were performed. The recoveries were definitely lower when H_2_O:EtOH was used, suggesting that EtOH partially eluted the adsorbed PAHs. Indeed, EtOH has been reported to be an efficient dissolution solvent for these compounds^33^. On the other hand, when pure H_2_O was used, the microparticles generated agglomerates, thus hindering an efficient washing. The best compromise was found by using a minimal volume of EtOH to disperse the microparticles (50 μL), followed by 1 mL of water. This procedure allowed the complete removal of the detected impurity (chromatographic peak under the detection limit) and provided the best recovery among the three tests for most PAHs (Fig S2). Still, recovery values were under 30% for lighter PAHs, while in the range 30–50% for the heavier ones. Therefore, to investigate the effect of the variables involved in the procedure and rationally optimize it, design of experiments and response surface methodology were employed.

### D-optimal design for the extraction protocol optimization

**Figure 1:**
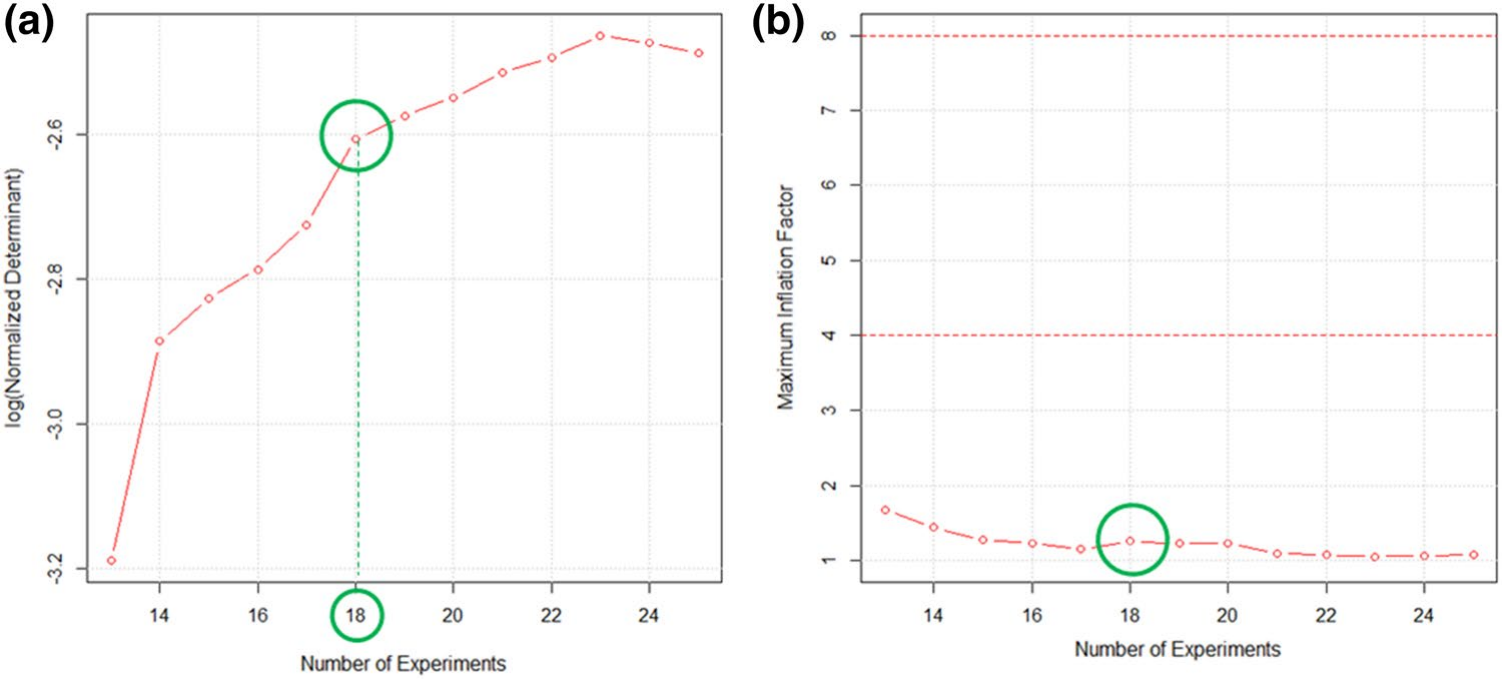
Graphs obtained by the D-optimal algorithm implementation on the candidate experiments: logarithmic graph of the normalized determinant of the information matrix (**a**) and maximum variance inflation factor (VIF) graph (**b**). The number of experiments selected is indicated by the green circle.

Several factors potentially influence the MagMIP dSPE procedure, making the multivariate approach the most effective way to reach an optimal response^22,25^. In those cases, the implementation of a screening design followed by a response surface design is generally advisable^34^. Indeed, with the first, the most important variables are identified, permitting to restrict the investigation to them. Then, a proper mathematical model which correlates these variables to the responses can be built. However, in our study, some considerations based on previous knowledge allowed us to narrow the list of factors to investigate. Theoretically, the following variables could influence the interaction of the PAHs with the MagMIP: V_matrix_/m_µp_ ratio, number of extractions, extraction time and mode. In addition, the solvent volume for back extraction (BE), type of solvent, number of the BEs, BE time and BE mode are likely to affect analytes desorption. Based on previous results obtained in our laboratory, all variables affecting desorption were disregarded. In fact, we supposed that, once the PAHs are sorbed onto the MIP through specific binding, the process become independent from the matrix from which they were extracted. The chosen BE solvent was acetone, since recommended by the manufacturer, while its volume and the number of BE were fixed at precautionary levels (namely two BE with a solvent mL/microparticles mg ratio of 0.075). BE time and mode were found not to have an effect on PAHs recovery^22^, due to the high affinity of this solvent for the target compounds, and were therefore set at the most convenient levels, corresponding to a 2 min vortex agitation. On the other hand, all the variables associated to the adsorption step were investigated (Table 1). These variables, characterized by a different nature (quantitative, semiquantitative and qualitative), could not be simultaneously studied by a conventional experimental design (such as central composite designs or Box-Behnken design)^34^. In such situations, the D-optimal design is the most suitable choice, since it allows to consider irregular experimental domains, different type of variables and to customize the experimental plan, by deciding the model coefficients which are necessary to build a satisfactory response curve. No previous knowledge was present regarding the interaction mode of the MagMIP microparticles with the viscous samples under consideration. Therefore, all computable coefficients were included in the postulated model. Linear and quadratic terms were necessary for the quantitative variables (X_1_ and X_2_), while only linear terms for X_3_ and X_4_. In addition, all possible interactions among factors were taken into account. The supposed response model (Eq. 1) served to calculate the necessary number of experiments (at least equal to the number of coefficients, namely 13). The optimal set of experiments was chosen among all possible candidate experiments (see paragraph 2.3), by selecting the ones which gave a good compromise between obtainable information and experimental effort. The postulated coefficients and the maximum number of tests were given as input in the CAT software, to obtain the logarithmic graph of the normalized determinant of the information matrix (Fig. 1a)^27^. Also, the maximum variance inflation factor (VIF) graph was obtained (Fig. 1b), which helps in choosing the points of the design. VIF are calculated for each coefficient by using the information matrix of a design and give information on the covariance among the coefficients (the lower, the better their estimation). The best VIFs are equal to 1, but good designs are still obtained if VIF is < 4.

By looking at the first graph (Fig. 1a), the best number of experiments should have been 23; still, by verifying the maximum VIF graph, also the minimum number of experiments would give a rather satisfactory estimation of the coefficients (max VIF < 2). A compromise was reached by selecting the solution with 18 experiments, for which the logarithm of the normalized determinant showed a significant leap, compared to the solutions with a lower number of experiments.

**Figure 2:**
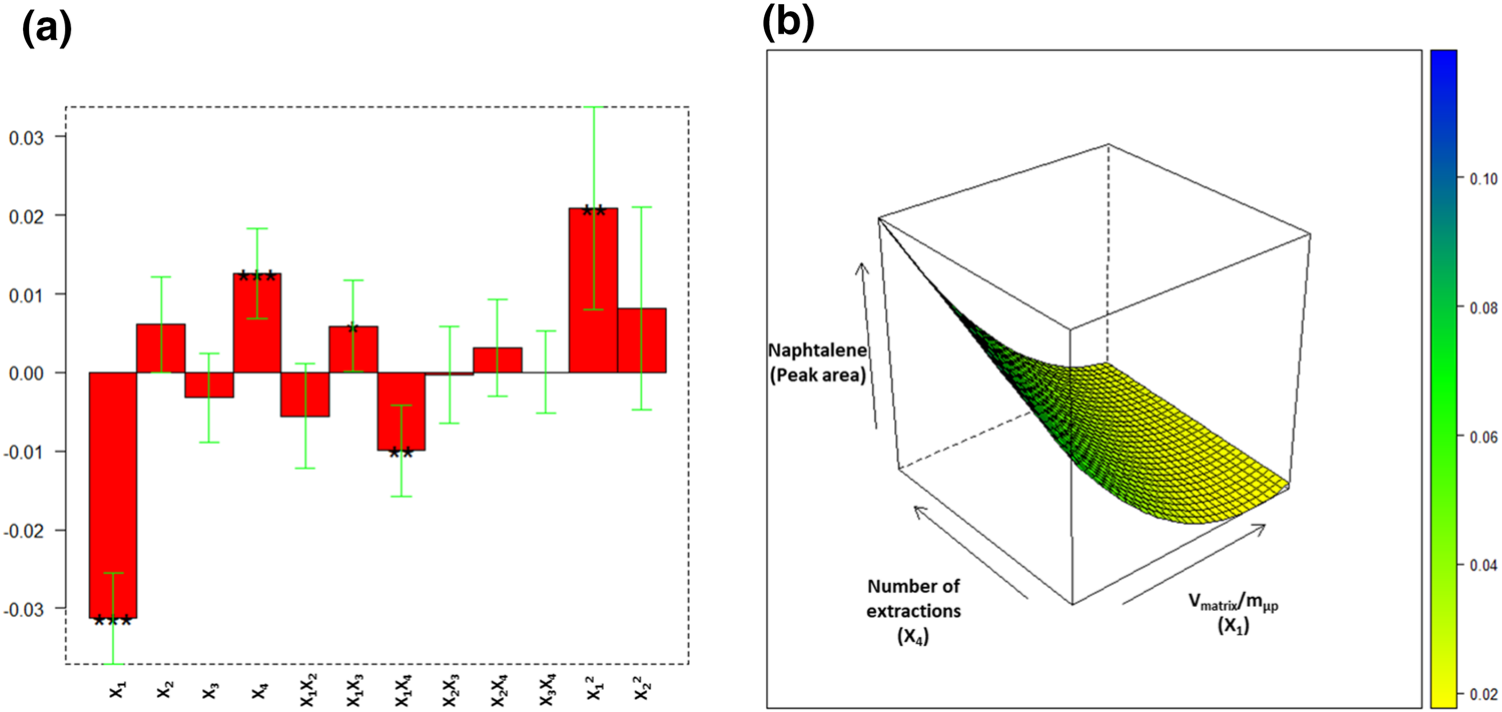
Coefficients’ plot for NAP model: significance level and sign of each computed coefficient (**a**); response surface for NAP model (**b**).

After performing the 21 experiments (18 + 3 replicates), the recoveries of the PAHs (responses) were used to compute the surface response curves. It must be noted that the variable X_1_, namely V_matrix_/m_µp_, was evaluated by keeping the mg of MagMIP constant and by changing the volume of solution. Therefore, in the different experiments, different initial spiked concentrations were used, in order to obtain the same absolute PAHs’ amount and therefore the same concentration in the final extract. By doing so, the recoveries of the 21 trials could be compared and used as responses for the models. The coefficients of all computed models are reported in Table S3, along with explained variances. Highly informative models were obtained for most analytes, with explained variances in the range 86–92% for PAHs with mass up to 202 Da and 75–80% for PAHs with mass up to 278 Da. The only two exceptions were BaA and BghiPE, which showed a lower, but still acceptable, explained variance (58% and 61%, respectively). Table S4 summarizes the significance information on all coefficients and their sign, namely the “direction” of the effect. It is worth highlighting that the responses for the 16 PAHs were highly correlated, and the most significant coefficients influenced all responses in the same way. As an example, Fig. 2a shows the coefficients’ plot for NAP model, which indicates the significance level and sign of each computed coefficient, while Fig. 2b reports the response surface, obtained by selecting the two most important factors as the axes. The response surfaces for BaA, CHR, BbF + BkF and BaPY are also reported in supplementary information (Figs. S3–S6).

All coefficient plots were quite similar, as well as the obtained response surfaces. The linear term of variables X_1_ (V_matrix_/m_µp_) resulted to have a significant negative influence on all responses, while the linear term of X_4_ (number of extractions) demonstrated a positive impact on all recoveries except for CHR, BaA and BghiPE. The fact that the number of extractions for these three analytes did not show a significant effect may be ascribed to their solubility in the initial matrix. Indeed, a lower solubility in EtOH:glycerin may favor the interaction with the MagMIP, thus providing good extraction efficiency even with a single extraction. However, for most PAHs, recovery improved by increasing the amount of MagMIP microparticles (for a fixed sample volume) and by using two consecutive extractions. The other significant coefficients (even though not for all responses) were the interactions among X_1_ (V_matrix_/m_µp_) and X_3_ (extraction mode), the interaction among X_1_ and X_4_ (number of extractions) and the quadratic term of X_1_. By looking at Fig. 2b, it can be easily deduced that the direction to maximize the response was toward lowering V_matrix_/m_µp_ ratio and performing a higher number of extractions.

### Further optimization and evaluation on real samples

The mathematical models obtained by the experimental design gave us the indications to maximize the analytes’ recoveries. Still, none of the performed experiments inside the chosen experimental domain provided satisfactory results (recoveries always under 50%). Therefore, while some of the factors were set at the best or most convenient values, two further tests were carried out in triplicate to evaluate lower V_matrix_/m_µp_ ratios, namely 0.2 and 0.1 mL/mg. Hence, 10 and 20 mg of MagMIP were used to perform an extraction on 2 mL of blank spiked matrix. Best results were obtained by using the highest amount of microparticles (20 mg of MagMIP), with recoveries in the range 40–80% and 60–120% for “light” and “heavy” PAHs, respectively. The only exception was NAP, which showed a recovery of approximately 30%. Throughout the whole optimization process, a clear division among the analytes was noted: the light PAHs, having a molecular mass lower than PYR (202 Da), generally exhibiting lower recoveries and the heavy PAHs, with a higher molecular mass, associated to higher recoveries. Indeed, MIP synthesized with PYR as template molecule has been reported to be more suitable to extract compounds with a higher molecular mass, probably due to the shape and chemical complementarity of the cavity. Kibechu et al. described the use of PYR-imprinted polymers for the extraction of NAP, AC, PYR, BaPYR, BkF from waters, indicating an approximately 70% recovery for NAP and AC, while quantitative for the others^35^.On the other hand, Garballo-Rubio et al. employed a commercial PYR-imprinted polymer for the extraction of BaA, CHR, BbF, BkF and BaP, while its use for lighter PAHs was not even tried^36^.

**Figure 3:**
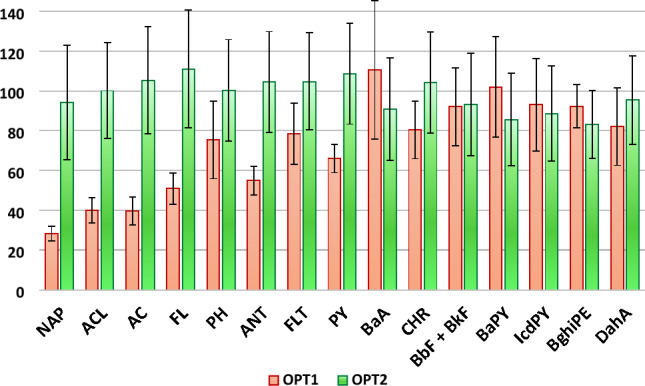
Comparison among recoveries (%) obtained by applying the optimized protocol on a spiked blank matrix (OPT1) and on a diluted real sample (OPT2).

After these latter tests, the final protocol (see paragraph 2.5) was applied to a pool of BDs, spiked with a known amount of the analytes, to verify the recovery on complex samples. Since interferences from the real matrix were highly probable, the BDs were diluted with H_2_O in a proportion of 3:1 (extract/water, w/w) before applying the MagMIP treatment. Six replicates were carried out on the BDs pool in different days, to verify the recovery on real samples as well as the intermediate precision of the overall method. Despite the complexity of the samples, the mean recovery values (76–100%) were higher than those obtained by using the same protocol applied to the spiked blank matrix (Fig. 3). This result suggests that the dilution with water prior processing had a favourable effect on the adsorption of the PAHs onto the MIP material.

### Analytical performances and greenness evaluation

The final method demonstrated good figures of merit for the quantitation of PAHs in the considered matrix, summarized in Table 3. Calibration curves were built by applying the internal standard approach and linearity was verified in the range LOQ-50 μg L^−1^ for most PAHs, while from LOQ to 25 μg L^−1^ for BaA, CHR, BbF, BkF and BaPY. Given the expected low concentrations, the ranges were considered fully acceptable. Acceptable specificity was attained by the application of the SIM MS acquisition mode and by checking retention times. Intra-day instrumental precision, expressed as relative standard deviation (RSD%) was ≤ 5% for all analytes; inter-day RSD% was below 12% for most analytes; the PAHs with the highest molecular mass showed a lower precision, with RSD% between 13 and 18%. LODs and LOQs were evaluated in real samples, by considering the noise signal adjacent to each peak. Despite the complexity of the sample, low noise signal was observed, leading to LODs and LOQs in the range 0.2–3.8 μg kg^−1^ and 0.7–12.6 μg kg^−1^, respectively.

**Table 3:** Analytical performances of the proposed magMIP- GC–MS method.

Compound	R^2^	Linearity (µg L^−1^)	Intra-day instrumental precision (RSD%)	Inter-day instrumental precision (RSD%)	LOD^a^ (µg kg^−1^)	LOQ^a^ (µg kg^−1^)	REC (%)	Intra-day procedural precision (n = 3) (RSD%)	Inter-day procedural precision (n_tot_ = 6) (RSD%)
NAP	0.999	1.6–50	1.5	1.8	0.4	1.5	94	2.1	30.7
ACL	0.998	1.4–50	2.5	3.3	0.4	1.4	100	7.7	24.0
AC	0.997	3.3–50	2.3	2.3	1.0	3.2	105	2.8	25.4
FL	0.991	0.9–50	1.4	2.7	0.3	0.9	111	3.1	26.5
PH	0.999	0.7–50	2.7	4.8	0.2	0.6	100	3.9	25.4
ANT	0.998	0.7–50	4.3	5.2	0.2	0.7	104	14.7	24.2
FLT	0.998	0.8–50	2.3	3.7	0.2	0.7	105	3.9	23.2
PY	0.995	0.7–50	2.9	4.3	0.2	0.7	109	3.2	23.3
BaA	0.983	2.7–25	4.6	11.9	0.8	2.6	91	1.9	28.5
CHR	0.989	2.4–25	3.3	3.7	0.7	2.3	104	2.5	24.3
BbF + BkF^b^	0.985	2.1–25	3.6	4.7	0.6	2.1	93	4.5	27.7
BaPY	0.973	4.9–25	3.6	7.4	1.4	4.8	86	1.5	27.2
IcdPY	0.997	2.4–50	4.5	16.3	0.7	2.3	89	15.5	26.9
DahA	0.993	1.4–50	5.1	13.6	0.4	1.4	95	16.1	23.3
BghiPE	0.997	13.0–50	4.4	18.4	3.8	12.6	83	5.5	20.6

^a^LODs and LOQs refer to the whole analytical method, considering the pre-concentration factor and matrix density.

^b^BbF + BkF indicates values for the two analytes together (partially co-eluted peaks).

As already mentioned, good average recoveries were obtained on a spiked real matrix, in the range 76–100%. The use of deuterated internal standards, added at the beginning of the sample processing, allowed to guarantee acceptable accuracy and precision. Indeed, thanks to this approach, the variability of the sample preparation as well as matrix effects were mostly compensated. By evaluating the procedural precision including IS at the beginning of the pre-treatment, the RSD% values were 1.5–7.7% for 13 analytes and approximately 15% for ANT, IcdPY and DahA. This result was probably due to the general lower sensitivity and instrumental precision for the latter compounds. As for the inter-day precision of the overall method, an RSD% in the range 20–30%, was observed for all analytes. These values were considered acceptable, since they encompass instrumental and inter-day procedural variability.

**Figure 4:**
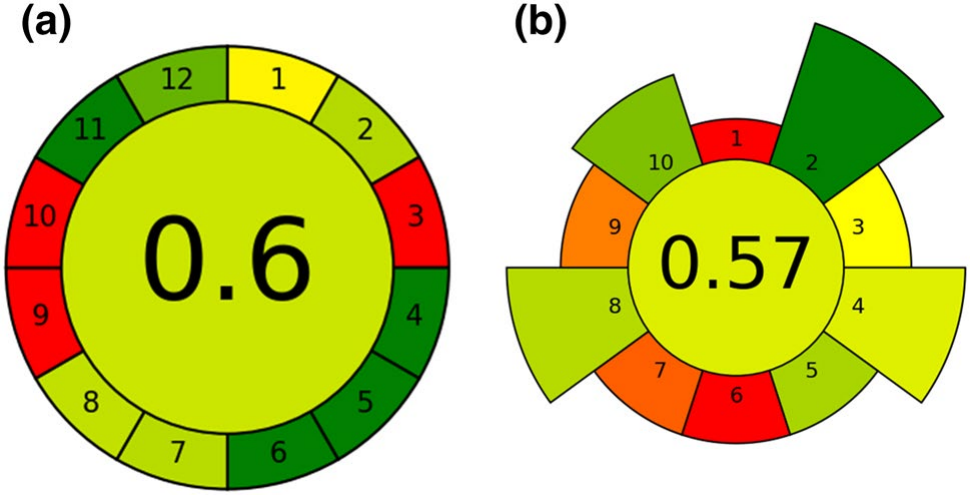
Pictograms obtained by the application of the AGREE (**a**) and AGREEprep (**b**) metrics.

The method was evaluated through two metrics based on the principles of green analytical chemistry (GAC), namely AGREE and its version specifically related to the sample preparation step (AGREEprep). In both cases an overall score of approximately 0.6/1 was gained, as shown in Fig. 4. This demonstrated that the method has a sufficient degree of eco-friendliness. Going in detail, when applying the AGREE software, each “sub-score” refers to one of the twelve GAC principles. Among them, the method received a low score in the principle 3, 9 and 10. The principle 3 was not fulfilled since it concerns the performance of in-situ measurements; principle 9 received a low score due to the use of GC-MS (high required energy); finally, principle 10 was not fulfilled due to the use of not renewable materials. Still, all other aspects of the method (waste production, sample size, use of hazardous reagents, number of discrete steps in the procedure…) gained a good score, indicated by the green colours. As far as AGREEprep is concerned, once again the sample processing performed in lab (ex-situ) and the use of complex instrumentation determined a bad evaluation in points number 1 and 9, respectively. In addition, points number 6 and 7 gained a low score due to the non-automation of sample treatment and the consequent low throughput, also related to the quite long GC-MS run time. However, it is worth noting that for the analysis of toxic substances such as PAHs, at very low concentration levels, the use of sensitive and specific instrumentation such as GC-MS is unavoidable. Considering that, the overall rating of the method was satisfactory.

### Analysis of the bud-derivatives samples

**Figure 5:**
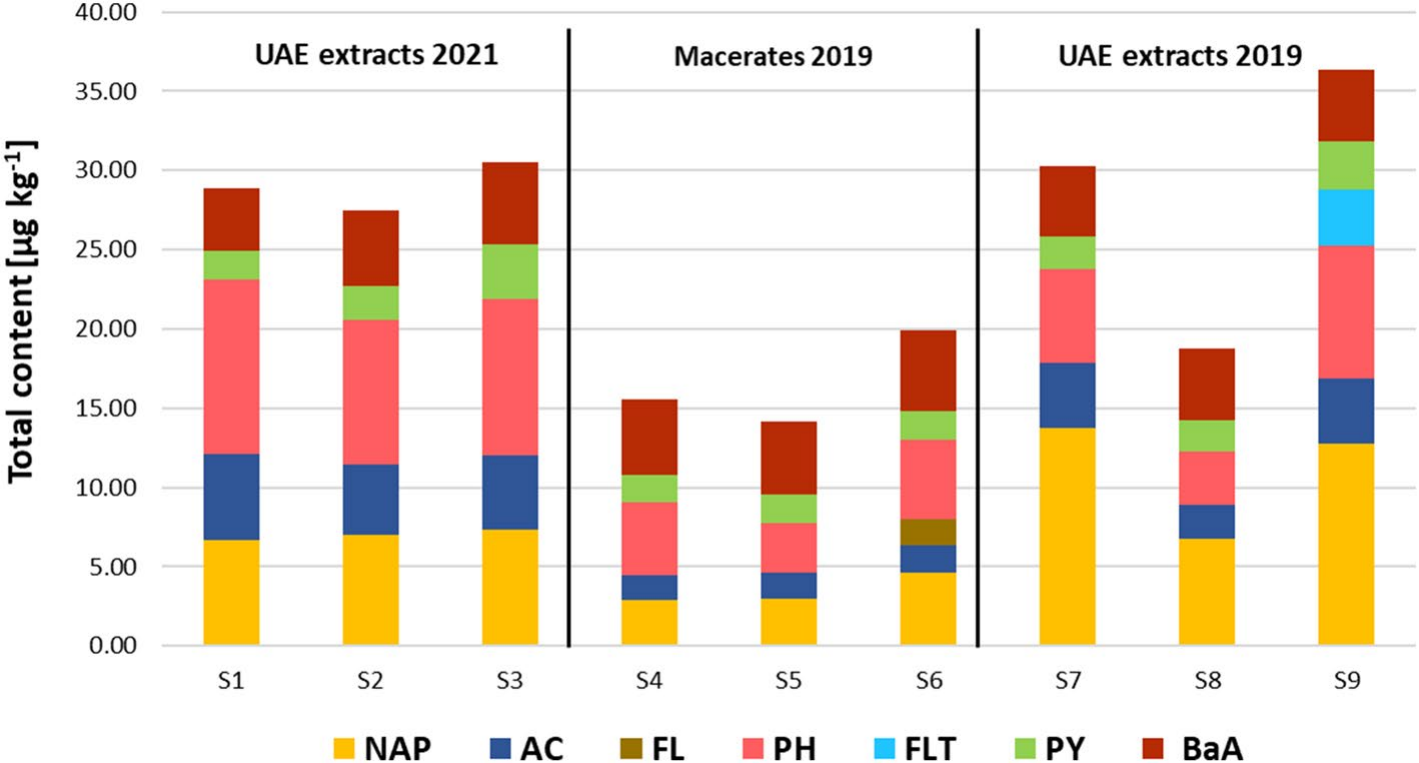
Total content of PAHs in the different samples. S1, S4 and S7: low pollution samples; S2, S5 and S8: medium pollution samples; S3, S6 and S9: high pollution samples (see Table S2 for more information).

The optimized method was applied to 9 BD samples. The analysis results, expressed in μg kg^−1^, are summarized in Fig. 5, while single concentrations are provided in Table S5 (Supplementary Information).

NAPH, AC, PH, PY and BaA were detected in all samples at concentrations ranging from 1.6 to 13.7 μg kg^−1^. In the samples obtained by maceration in 2019 (S4, S5 and S6), the PAH concentrations were in most cases lower than those found in the samples obtained by sonication in 2019 and 2021. This result could be likely explained by the fact that the maceration technique, generally less drastic than sonication, is also less effective in extracting any pollutant present in the raw material. An exception was represented by BaA, which instead showed concentrations in the same range as the samples obtained by sonication. FL was detected in all samples, but quantitation was possible only in S6—one of the “high pollution” samples- at 1.6 μg kg^−1^. A similar case was represented by FLT, detected below LOQ in all samples, except sample 9, where a low concentration was determined (3.5 μg kg^−1^). CHR, BbF and BkF were detected just above LOD in all samples but were not quantifiable.

As a general remark, the highest concentration of PAHs was always found in the sample with the highest degree of pollution, but this trend was not reported from low to medium pollution samples, whose concentrations were not significantly different. Among the samples obtained by sonication, the PAHs’ concentrations were slightly lower in 2019 than in 2021, except for NAP.

It is worth noting that the PAHs present in the BDs were mainly those characterized by a medium–low molecular mass. This is probably due to the higher solubility of these analytes in the ethanol:glycerin mixture used during derivatives preparation. The detection of these “lighter” PAHs in the considered samples implies a slight contamination of the collected buds. The reason may be attributed to the fact that the buds were taken in early spring, when the temperatures are not particularly high in the considered Italian region (15–20 °C). Indeed, the vegetation-atmosphere distribution of PAHs also depends on the ambient temperature: at low temperature (as in spring) the PAHs in the gaseous phase are preferentially distributed on the waxy surface of leaves and buds, while at higher temperatures, such as in summer, some PAHs volatilize and return to the atmosphere^37^.

It is possible to state that minimal concentrations of PAHs were present in the analyzed BDs. Still, the found levels do not cause particular concern. In fact, the only regulated PAH detected was BaA, but its concentration (approximately 5 μg kg^−1^ in all samples) was far below the EC legal limit (50 μg kg^−1^ for the sum of BaA, BaPYR, BbF and CHR). On the other hand, the analyte which presented the highest concentration (close to 14 μg kg^−1^) was NAP, which is also the least toxic. From this perspective, buds deriving from city pruning could be an interesting alternative to those collected in spontaneous trees from less polluted areas; their employment in the production of nutraceutical products would be desirable, according to the principle of circular economy.

## Conclusions

In the present work, an eco-sustainable extraction technique was evaluated to determine the presence of the 16 priority PAHs in bud-derived extracts. The extraction technique used, based on d-SPE, employed a specific solid phase: magnetic microparticles covered by a molecular imprinted polymer. By applying the chemometric technique D-Optimal Design, it was possible to rapidly optimize the sample preparation procedure, which, combined with GC-MS analysis, showed optimal recoveries, satisfactory LOD and LOQ values in matrix and acceptable overall precisions. The optimized method allowed a simple and green PAHs extraction procedure, with a consumption of very low volumes of acetone. The applicability was demonstrated by the analysis of several BDs samples, deriving from bud collected in different areas of the Italian city of Turin, whose contamination levels were below the PAHs concentration limits posed by the European Commission for food supplements.

### Supplementary Information

Supplementary Information.
