# Supplementary material for: Determination of polycyclic aromatic hydrocarbons in bud-derived supplements by magnetic molecular imprinted microparticles and GC-MS: D-optimal design for a fast method optimization
Source: Sci Rep. 2023 Oct 16;13:17544. doi: 10.1038/s41598-023-44398-8 (PMC10579315; doi:10.1038/s41598-023-44398-8)
Supplement: Supplementary file 1 — Supplementary Information. [file 41598_2023_44398_MOESM1_ESM.docx]

**Supplementary material**

**Determination of polycyclic aromatic hydrocarbons in bud-derived supplements by magnetic molecular imprinted microparticles and GC-MS: D-optimal design for a fast method optimization**

Barbara Benedetti^1^, Arianna Tronconi^1^, Federica Turrini^2^, Marina Di Carro^1^, Dario Donno^3^, Gabriele Loris Beccaro^3^, Raffaella Boggia^2^, Emanuele Magi^1^*

*^1^ Department of Chemistry and Industrial Chemistry, University of Genoa, Via Dodecaneso 31, 16146 Genoa, Italy*

*^2^ Department of Pharmacy, University of Genoa, Viale Cembrano 4, 16148 Genoa, Italy*

*^3^Department of Agriculture, Forestry and Food Science, University of Turin, Largo Braccini 2, 10095, Grugliasco (TO), Italy*

*Corresponding author. Tel.: +39 010 3536187; fax: +39 010 3536190. E-mail address: [emanuele.magi@unige.it](mailto:emanuele.magi@unige.it), Department of Chemistry and Industrial Chemistry, University of Genoa, Via Dodecaneso 31, 16146 Genoa, Italy

**Table of content:**

**Table S1: GC-MS information on the 16 analytes and relative internal deuterated standards**

**Table S2: details on the BDs samples**

**Fig. S1: tentative GC-MS identification of an impurity in the extract**

**Fig. S2: recoveries of different washing procedures**

**Table S3: coefficients of all computed models**

**Fig. S3-S6: response surface for BaA, CHR, BbF+BkF and BaPY models**

**Table S4: summary of the significance information of the models**

**Table S5: PAH content in BDs**

**Table S1**: Selected ions for SIM detection and retention times of the 16 PAHs under study and the 5 internal standards.

| Compound | Selected ion in SIM mode  (m/z) | Retention time  (min) | Assigned internal standard |
| --- | --- | --- | --- |
| Napthatlene (NAP) | 128 | 9.11 | d-NAP |
| acenaphthylene (ACL) | 152 | 12.84 | d-AC |
| acenaphthene (AC) | 154 | 13.26 | d-AC |
| fluorene (FL) | 166 | 14.50 | d-PH |
| phenanthrene (PH) | 178 | 16.76 | d-PH |
| anthracene (ANT) | 178 | 16.89 | d-PH |
| fluoranthene (FLT) | 202 | 19.60 | d-CHR |
| pyrene (PY) | 202 | 20.12 | d-CHR |
| benzo[a]anthracene (BaA) | 228 | 23.00 | d-CHR |
| chrysene (CHR) | 228 | 23.08 | d-CHR |
| benzo[b]fluoranthene (BbF)* | 252 | 25.66 | d-PE |
| benzo[k]fluoranthene (BkF)* | 252 | 25.74 | d-PE |
| benzo[a]pyrene (BaPY) | 252 | 26.55 | d-PE |
| indeno[1,2,3-c,d]pyrene (IcdPY) | 276 | 30.58 | d-PE |
| dibenzo[a,h]anthracene (DahA) | 278 | 30.72 | d-PE |
| benzo[g,h,i]perylene (BghiPE) | 276 | 31.37 | d-PE |
| Internal standards | | | |
| d-Napthatlene (d-NAP) | 136 | 9.06 |  |
| d-acenaphthene (d-AC) | 164 | 13.19 |  |
| d-phenanthrene (d-PH) | 188 | 16.71 |  |
| d-chrysene (d-CHR) | 240 | 23.03 |  |
| d-perylene (d-PE) | 264 | 26.74 |  |

* Benzo[b]fluoranthene and benzo[k]fluoranthene peaks were partially coeluted, thus they were considered as a unique peak.

**Table S2**: details on the BDs samples analyzed in this study.

| **SAMPLE NAME** | **Year** | **Extraction method** | **Pollution** | **Origin**  Turin (Italy) |
| --- | --- | --- | --- | --- |
| S1 | 2021 | UAE | Low | Superga Hill |
| S2 | 2021 | UAE | Medium | Ignazio Michelotti Park |
| S3 | 2021 | UAE | High | Massimo D'Azeglio Avenue |
| S4 | 2019 | Maceration | Low | Superga Hill |
| S5 | 2019 | Maceration | Medium | Ignazio Michelotti Park |
| S6 | 2019 | Maceration | High | Massimo D'Azeglio Avenue |
| S7 | 2019 | UAE | Low | Superga Hill |
| S8 | 2019 | UAE | Medium | Ignazio Michelotti Park |
| S9 | 2019 | UAE | High | Massimo D'Azeglio Avenue |


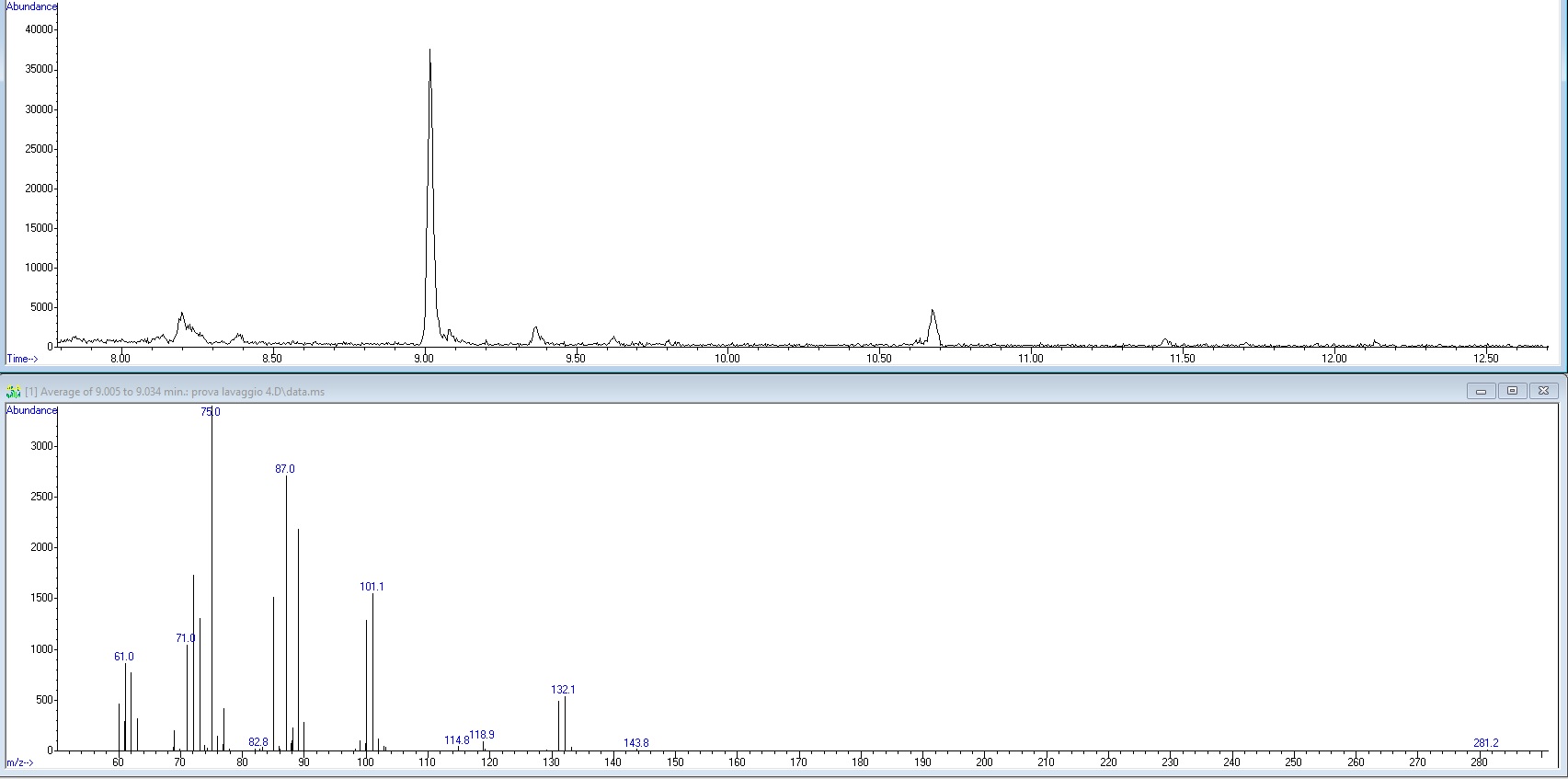

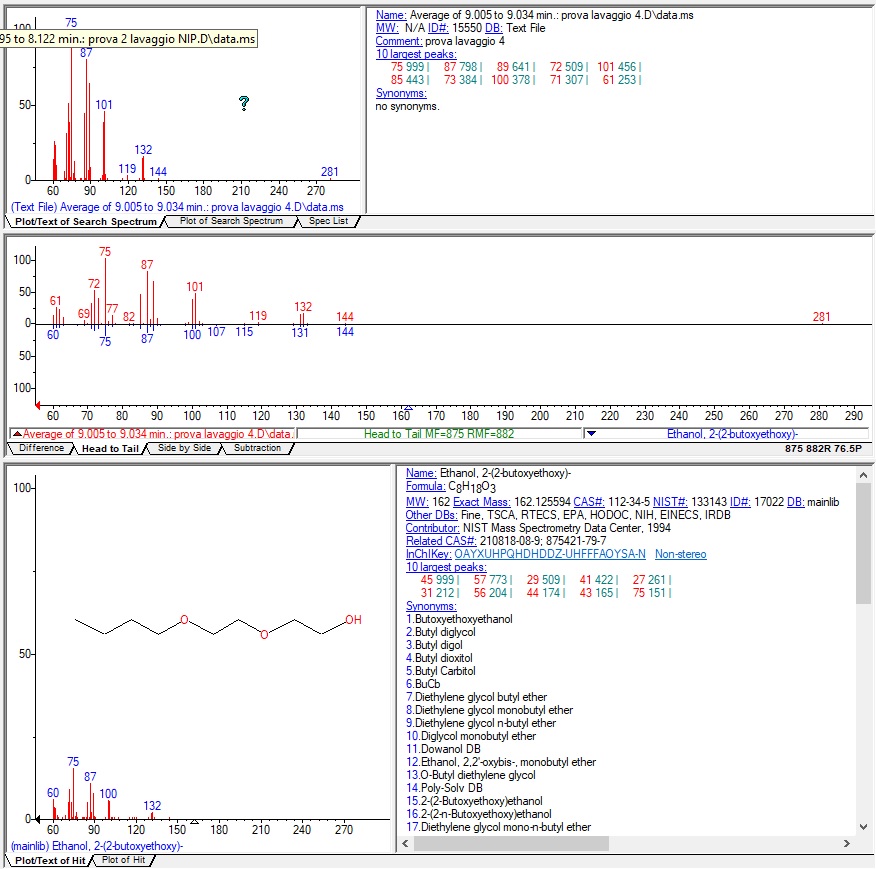


**Fig. S1**: chromatogram and mass spectrum of the peak at retention time 9.02 min. The NIST library tentatively identified the compound as 2-(2-Butoxyethoxy)ethanol.

**Fig. S2**: MagMIPs recoveries: washing procedures comparison. W1: 1 mL of H_2_O:EtOH (50:50, v/v); W2: 1 mL of H_2_O; W3: 50 μL EtOH + 1 mL H_2_O.

**Table S3**: coefficients and explained variance of all mathematical models computed from the results of the experimental design.

| Compound | Coefficients | | | | | | | | | | | | | Explained Variance (%) |
| --- | --- | --- | --- | --- | --- | --- | --- | --- | --- | --- | --- | --- | --- | --- |
|  | b_0_ | X_1_ | X_2_ | X_3_ | X_4_ | X_1_*X_2_ | X_1_*X_3_ | X_1_*X_4_ | X_2_*X_3_ | X_2_*X_4_ | X_3_*X_4_ | X_1_^2^ | X_2_^2^ |  |
| NAP | 0.0359 | -0.0312 | 0.0061 | -0.0032 | 0.0126 | -0.0055 | 0.0059 | -0.0100 | -0.0030 | 0.0031 | -0.000 | 0.0209 | 0.0082 | 92.43 |
| ACL | 0.0305 | -0.0270 | 0.0048 | -0.0023 | 0.0116 | -0.0033 | 0.0033 | -0.0071 | 0.0002 | 0.0034 | 0.0009 | 0.0217 | 0.0033 | 85.89 |
| AC | 0.0251 | 0.0223 | 0.0044 | -0.0010 | 0.0093 | -0.0027 | 0.0024 | -0.0058 | 0.0002 | 0.0027 | 0.0010 | 0.0165 | 0.0025 | 91.25 |
| FL | 0.0350 | -0.0296 | 0.0051 | -0.0014 | 0.0126 | -0.0038 | 0.0020 | -0.0086 | 0.0012 | 0.0040 | 0.0017 | 0.0213 | 0.0034 | 90.56 |
| PH | 0.0673 | -0.0368 | 0.0074 | -0.0038 | 0.0112 | -0.0036 | 0.0092 | -0.0065 | -0.0003 | 0.0051 | 0.0041 | 0.0370 | -0.0032 | 88.51 |
| ANT | 0.0766 | -0.0494 | 0.0139 | 0.0025 | 0.0198 | -0.0043 | -0.0040 | -0.0113 | -0.0034 | 0.0056 | 0.0021 | 0.0332 | 0.0121 | 86.88 |
| FLT | 0.0964 | -0.0573 | 0.0115 | -0.0014 | 0.0189 | -0.0023 | 0.0064 | -0.0039 | 0.0017 | 0.0094 | 0.0051 | 0.0490 | 0.0007 | 87.35 |
| PY | 0.1153 | -0.0698 | 0.0152 | -0.0005 | 0.0025 | -0.0037 | 0.0071 | -0.0074 | 0.0017 | 0.0126 | 0.0069 | 0.0612 | -0.0000 | 86.28 |
| BaA | 0.0220 | -0.0168 | 0.0033 | -0.0013 | 0.0034 | 0.0001 | 0.0067 | -0.0002 | 0.0010 | 0.0035 | 0.0046 | 0.0211 | -0.0057 | 58.16 |
| CHR | 0.1083 | -0.0646 | 0.0126 | -0.0023 | 0.0164 | 0.0001 | 0.0116 | -0.0021 | 0.0020 | 0.0108 | 0.0073 | 0.0682 | -0.0056 | 79.18 |
| BbF + BkF | 0.1345 | -0.0724 | 0.0212 | 0.0043 | 0.0244 | 0.0010 | 0.0080 | -0.0059 | -0.0014 | 0.0112 | 0.0123 | 0.0722 | -0.0052 | 78.14 |
| BaPY | 0.0520 | -0.0247 | 0.0092 | 0.0019 | 0.0099 | 0.0017 | -0.0011 | -0.0047 | -0.0014 | 0.0052 | 0.0016 | 0.0257 | 0.0004 | 76.46 |
| IcdPY | 0.0358 | -0.0192 | 0.0058 | 0.0042 | 0.0084 | 0.0013 | 0.0001 | -0.0016 | -0.0009 | 0.0037 | 0.0049 | 0.0171 | -0.0041 | 75.11 |
| DahA | 0.0414 | -0.0239 | 0.0094 | 0.0035 | 0.0111 | -0.0019 | -0.0000 | -0.0047 | -0.0011 | 0.0060 | 0.0046 | 0.0198 | -0.0018 | 80.64 |
| BghiPE | 0.0299 | -0.0172 | 0.0050 | 0.0005 | 0.0070 | 0.0031 | -0.0012 | -0.0018 | -0.0006 | 0.0024 | 0.0034 | 0.0177 | -0.0013 | 61.3 |


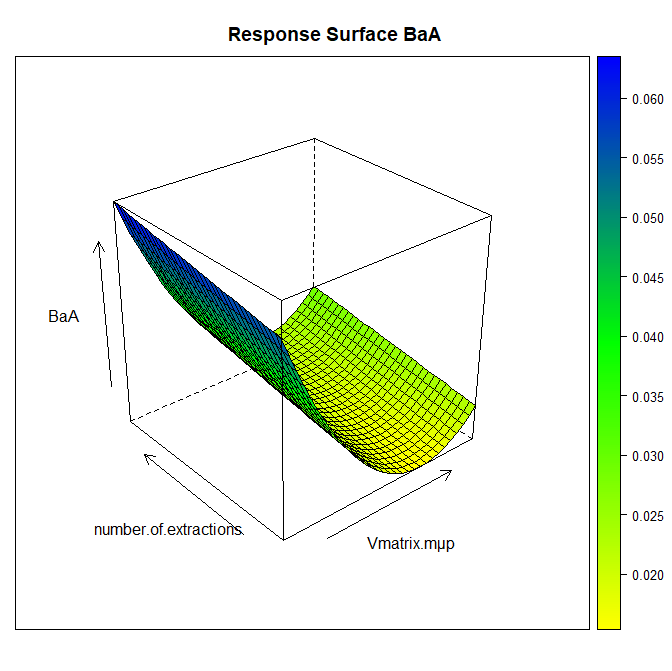


**Fig. S3**: response surface for BaA model.


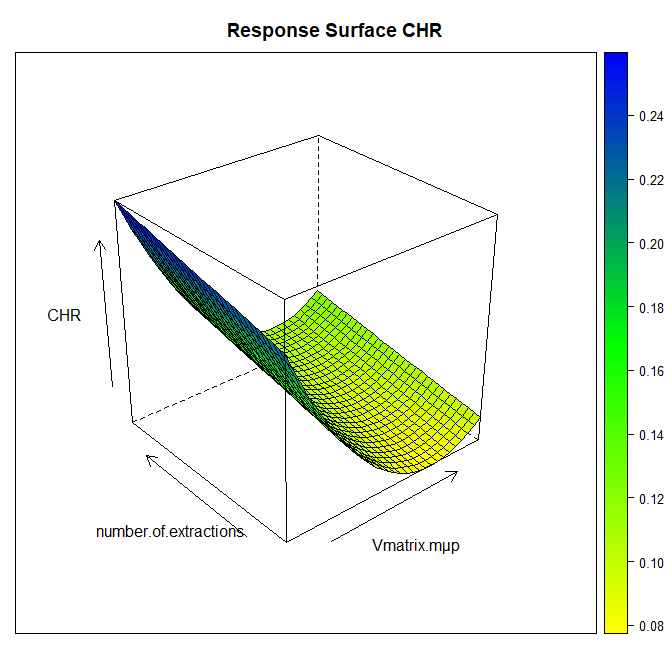


**Fig. S4**: response surface for CHR model.


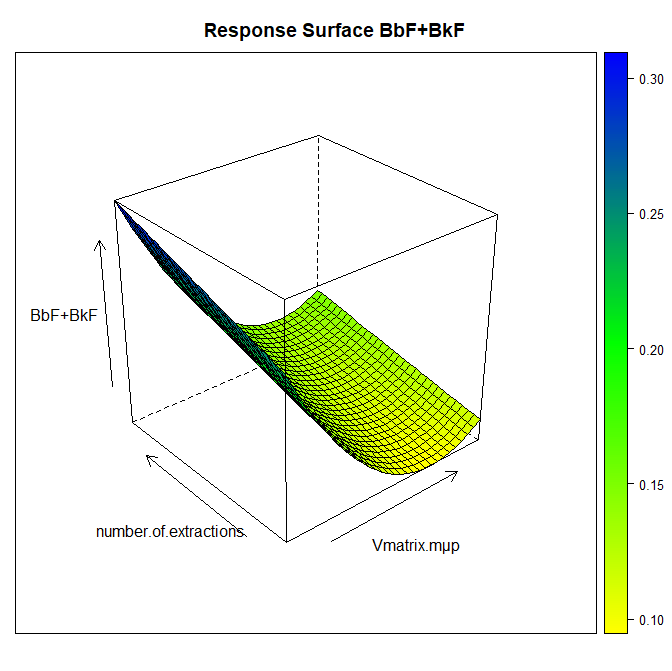


**Fig. S5**: response surface for BbF+BkF model.


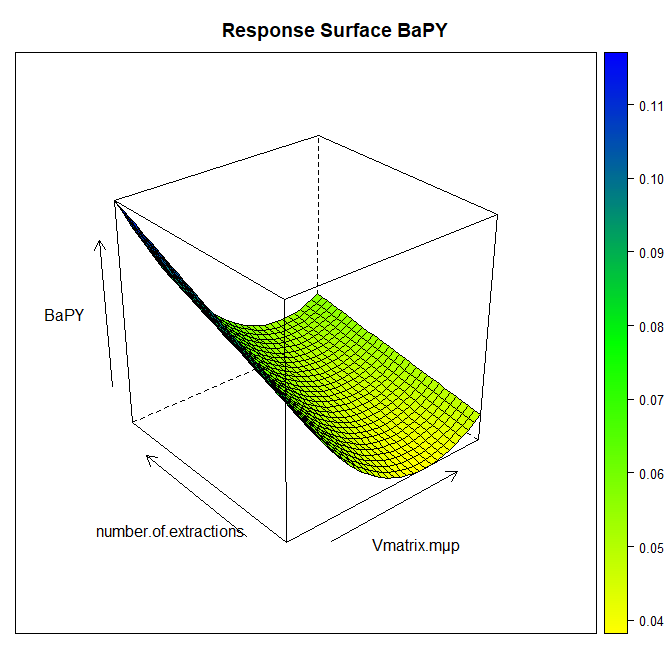


**Fig. S6**: response surface for BaPY model.

**Table S4:** details on the single models correlating the GC-MS peak areas and the factors considered in the D-optimal design. Significance level and sign is indicated for each model coefficient.

| Compound | V_matrix_/m_µp_ (X_1_) | | Extraction time (X_2_) | | Number of extractions (X_4_) | | Interaction X_1_X_3_ | | Interaction X_1_X_4_ | | Quadratic term X_1_^2^ | |
| --- | --- | --- | --- | --- | --- | --- | --- | --- | --- | --- | --- | --- |
|  | significance level ^a^ | sign | significance level | sign | significance level | sign | significance level | sign | significance level | sign | significance level | sign |
| NAP | *** | - | ns |  | *** | + | * | + | ** | - | ** | + |
| ACL | *** | - | * | + | *** | + | ns |  | ** | - | *** | + |
| AC | *** | - | ns |  | ** | + | ns |  | * | - | ** | + |
| FL | *** | - | ns |  | ** | + | ns |  | * | - | ** | + |
| PH | *** | - | ns |  | * | + | * | + | ns |  | ** | + |
| ANT | *** | - | * | + | ** | + | ns |  | ns |  | * | + |
| FLT | *** | - | ns |  | ** | + | ns |  | ns |  | ** | + |
| PY | *** | - | ns |  | * | + | ns |  | ns |  | ** | + |
| BaA | ** | - | ns |  | ns |  | ns |  | ns |  | * | + |
| CHR | *** | - | ns |  | ns |  | ns |  | ns |  | ** | + |
| BbF + BkF ^b^ | *** | - | ns |  | * | + | ns |  | ns |  | * | + |
| BaPY | *** | - | ns |  | * | + | ns |  | ns |  | * | + |
| IcdPY | *** | - | ns |  | * | + | ns |  | ns |  | * | + |
| DahA | *** | - | * | + | ** | + | ns |  | ns |  | * | + |
| BghiPE | ** | - | ns |  | ns |  | ns |  | ns |  | * | + |

1. *=p<0.05; **=p<0.01; ***=p<0.001
2. Partially coeluted peaks, thus only one model computed

**Table S5**: Results of the analyses on bud derivatives coming from areas subjected to different anthropic impact.

| Compound | S 1 | S 2 | S 3 | S 4 | S 5 | S 6 | S 7 | S 8 | S 9 |
| --- | --- | --- | --- | --- | --- | --- | --- | --- | --- |
| µg kg ^-1^ | | | | | | | | | |
| NAP | 6.7 ± 0.1 | 7 ± 0.1 | 7.3 ± 0.2 | 2.89 ± 0.06 | 2.99 ± 0.06 | 4.6 ± 0.1 | 13.7 ± 0.3 | 6.8 ± 0.1 | 12.8 ± 0.3 |
| ACL | <LOQ | <LOQ | <LOQ | <LOQ | <LOQ | <LOQ | <LOQ | <LOQ | <LOQ |
| AC | 5.4 ± 0.2 | 4.4 ± 0.1 | 4.7 ± 0.1 | 1.59 ± 0.04 | 1.65 ± 0.05 | 1.76 ± 0.05 | 4.2 ± 0.1 | 2.08 ± 0.06 | 4.1 ± 0.1 |
| FL | <LOQ | <LOQ | <LOQ | <LOQ | <LOQ | 1.64 ± 0.05 | <LOQ | <LOQ | <LOQ |
| PH | 11 ± 0.4 | 9.1 ± 0.4 | 9.8 ± 0.4 | 4.6 ± 0.2 | 3.1 ± 0.1 | 5 ± 0.2 | 5.9 ± 0.2 | 3.4 ± 0.1 | 8.4 ± 0.3 |
| ANT | <LOD | <LOD | <LOD | <LOD | <LOD | <LOD | <LOD | <LOD | <LOD |
| FLT | <LOQ | <LOQ | <LOQ | <LOQ | <LOQ | <LOQ | <LOQ | <LOQ | 3.5 ± 0.1 |
| PY | 1.77 ± 0.06 | 2.19 ± 0.07 | 3.4 ± 0.1 | 1.7 ± 0.05 | 1.85 ± 0.06 | 1.81 ± 0.06 | 2.02 ± 0.06 | 1.95 ± 0.06 | 3 ± 0.1 |
| BaA | 3.95 ± 0.08 | 4.73 ± 0.09 | 5.2 ± 0.1 | 4.78 ± 0.09 | 4.55 ± 0.09 | 5.1 ± 0.1 | 4.5 ± 0.09 | 4.53 ± 0.09 | 4.58 ± 0.09 |
| CHR | <LOQ | <LOQ | <LOQ | <LOQ | <LOQ | <LOQ | <LOQ | <LOQ | <LOQ |
| BbF + BkF | <LOD | <LOQ | <LOQ | <LOQ | <LOQ | <LOQ | <LOQ | <LOQ | <LOQ |
